# Computational Approaches to Molecular Properties, Chemical Reactivity, and Drug Virtual Screening

**DOI:** 10.3390/molecules25225301

**Published:** 2020-11-13

**Authors:** Alessandro Ponti

**Affiliations:** Istituto di Scienze e Tecnologie Chimiche “Giulio Natta”, Consiglio Nazionale delle Ricerche (SCITEC-CNR), via C. Golgi 19, 20,133 Milano, Italy; alessandro.ponti@scitec.cnr.it

In the first paragraph of his 1929 paper “Quantum Mechanics of Many-Electron Systems”, Dirac wrote that “The underlying physical laws necessary for the mathematical theory of a large part of physics and the whole of chemistry are thus completely known, and the difficulty is only that the exact application of these laws leads to equations much too complicated to be soluble. It therefore becomes desirable that approximate practical methods of applying quantum mechanics should be developed, which can lead to an explanation of the main features of complex atomic systems without too much computation” [1]. Notwithstanding that the presently available computational power is enormously larger than in 1929, this passage still retains full validity. Huge amounts of ingenuity and effort have been spent by scientists for 90 years and highly impressive achievements have been earned in both directions indicated by Dirac, that is, improvement of approximate methods and insight into chemistry. That is why computational and theoretical chemistry is still today a very active, competitive, fast-changing, and intriguing research field.

The “Computational and Theoretical Chemistry” section of *Molecules* (Section Editor-in-Chief, Dr. James W. Gauld) provides an open-access platform for the publication of original papers and review articles on the development of computational and theoretical chemistry and its application to unravelling chemical phenomena. Of course, computational and theoretical chemistry is not limited to the many flavors of quantum chemistry, e.g., applications to chemistry of revered thermodynamics and sparkling artificial intelligence (just to name two) contribute to the understanding of chemistry. This section, which was established in 2010 and since published 255 papers, invites original articles and critical reviews in the field of computational and theoretical chemistry, including (but not limited to) the development of theoretical approaches and computational methods and the application of computational chemistry to provide insight into important phenomena and problems in chemistry and biochemistry.

To give a flavor of the exciting research that is being published in *Molecules*, we here highlight some recent papers in the fields of molecular properties, chemical reactivity, and biologically relevant interactions. Amador-Balderas et al. [2] used the conceptual and computational framework of density functional theory (DFT) to analyze how substituents affect the acidity of benzoic acids and found that the substituent effect of electron-releasing substituents (which decrease the acidity) increases as *ortho* > *meta* > *para***,** and vice versa for electron-withdrawing substituent (which increase the acidity). 

An in-depth study of the chemical bonding in Li and Na adsorbates on pristine and defective graphene and graphene oxide has been carried out by Dimakis et al. [3] They proved the importance of defects for this adsorption processes and showed that both metal–carbon and metal–metal bonds have a definite covalent character. 

A central concept in organic chemistry, i.e., aromaticity, has been used by Woller et al. [4] to investigate the structure-property relationships of UV/Vis absorption and non-linear optical properties of a series of Hückel porphyrinoids. A detailed analysis of the absorption bands in terms of polarization, intensity, splitting and composition allowed them to conclude that aromaticity dictates the photophysical properties of Hückel porphyrinoids, whereas it is not the only factor determining the magnitude of the non-linear optical properties.

Reactivity is of course a central theme in chemistry though it unfortunately requires high theory levels and large computational resources to be tackled properly. It is therefore not surprising that a large fraction of recent papers deals with molecular reactivity. The Molecular Electron Density Theory (MEDT) is an interpretative tool to explain organic chemical reactivity. The paper by the MEDT developers Ríos-Gutiérrez and Domingo, and coworkers [5] investigated the [3+2] cycloaddition reaction of diphenyl nitrilimine and phenyl nitrile oxide to (*R*)-(–)-carvone, the molecule responsible for the flavor of caraway, dill and spearmint. After establishing the different reactive nature of diphenyl nitrilimine and phenyl nitrile oxide, the authors studied in depth all of the possible reactive channels and were able to account for the experimental outcome of these reactions and shed light on the reaction mechanism. 

Pyrolysis is a very important reaction class from the practical point of view. The computational modelling of pyrolysis is unfortunately complex because of the many reactive channels and the presence of radical species including, of course, triplet dioxygen. Tao et al. [6] conducted an experimental and computational study of the pyrolysis of 1,1,1,4,4,4-hexafluoro-2-butene possibly leading to the production of dangerous hydrogen fluoride. Several reaction pathways were explored to study the formation mechanism of hydrogen fluoride, which turned out to be produced through intramolecular elimination and abstraction reactions. 

Drugs are primary targets of synthetic organic chemistry and their effective preparation is of utmost importance. A key ingredient of the latter is the absence of isomeric undesired products. Domingo at al. [7] studied the selectivity of the synthesis of an anthelminthic (and possibly anticancer) spiroisoxazoline drug derived from α-santonin. They could account for the total *ortho* regioselectivity and *syn* diastereofacial selectivity involving the exocyclic C–C double bond and explain the electronic interactions responsible for the selectivity. 

*Molecules* also welcomes expert critical reviews. A good example is the review by Nifant’ev and Ivchenko, [8] who reviewed the intriguing and mechanistically complex field of ring-opening polymerization (ROP) of cyclic esters (lactones, lactides, cyclic carbonates and phosphates). Despite that a partial rationalization of the seemingly diverse and cumbersome mechanisms of ROP catalyzed by metal complexes can be carried out within the framework of the coordination-insertion mechanism, their conclusion is that there is no comprehensive model of the ROP mechanism because of the huge variety of catalysts and ROP substrates. Nevertheless, some fundamental results have been obtained such as a computational methodology to find the reaction pathways based on ligand environment and ring-opening product coordination.

The computational modelling of intermolecular interactions is a very active field, mainly aimed at the discovery of new drugs and the understanding of their biochemical action. Studies in this field often rely on molecular-dynamics-based docking studied and QSAR for screening promising molecules. A review on the applications of computational methods in drug screening and design by Lin et al. [9] recently appeared in *Molecules*. 

Zhao et al. [10] screened aryl benzamide derivatives as negative allosteric modulators (NAMs) of mGluR5, a glutamate receptor that is an important target for the treatment of depression. Using a combination of 3D-QSAR (quantitative structure-activity relationship), molecular docking, and MD simulations, the relationship between the structure of 106 newly synthesized mGluR5-NAMs and their activity were explored. The binding pattern and mechanism were identified to provide guidance for the synthesis of new glutamate-related drugs. 

Autotaxin is an interesting drug target for the therapy of several diseases. Ren et al. [11] discovered nine new potentially-potent autotaxin inhibitors by a combinatory virtual screening procedure comprising crystallography-derived pharmacophore modelling, docking study, and QSAR analysis.

*Molecules* compare favorably with competing journals. It has a 2019 IF of 3.267, well above the median IF = 2.405 of the “Chemistry, Multidisciplinary” journals and the median IF = 2. 845 of the “Chemistry, Physical” journals (InCites), and lies in the Q2 of the “Chemistry, Multidisciplinary” (JCR category rank) The 2019 average publication time in *Molecules* is 14 days from submission to the first decision and about 3.5 days from acceptance to publication. *Molecules* published 4667 papers in 2019 with a rejection rate of 54%. The short publishing time, while maintaining a rigorous peer-review selection process, is proof of the high standards of the Editors and the Editorial Team of *Molecules*. We hope that you will consider submitting to *Molecules* your next paper in the field of Computational and Theoretical Chemistry.
